# Molecular Insights into Frataxin-Mediated Iron Supply for Heme Biosynthesis in *Bacillus subtilis*

**DOI:** 10.1371/journal.pone.0122538

**Published:** 2015-03-31

**Authors:** Andreas Mielcarek, Bastian Blauenburg, Marcus Miethke, Mohamed A. Marahiel

**Affiliations:** 1 Philipps-University Marburg, Department of Chemistry/Biochemistry, Marburg, Germany; 2 Department of Chemistry and Biochemistry, University of California Los Angeles, Los Angeles, California, United States of America; King's College London, UNITED KINGDOM

## Abstract

Iron is required as an element to sustain life in all eukaryotes and most bacteria. Although several bacterial iron acquisition strategies have been well explored, little is known about the intracellular trafficking pathways of iron and its entry into the systems for co-factor biogenesis. In this study, we investigated the iron-dependent process of heme maturation in *Bacillus subtilis* and present, for the first time, structural evidence for the physical interaction of a frataxin homologue (Fra), which is suggested to act as a regulatory component as well as an iron chaperone in different cellular pathways, and a ferrochelatase (HemH), which catalyses the final step of heme *b* biogenesis. Specific interaction between Fra and HemH was observed upon co-purification from crude cell lysates and, further, by using the recombinant proteins for analytical size-exclusion chromatography. Hydrogen–deuterium exchange experiments identified the landscape of the Fra/HemH interaction interface and revealed Fra as a specific ferrous iron donor for the ferrochelatase HemH. The functional utilisation of the *in vitro*-generated heme *b* co-factor upon Fra-mediated iron transfer was confirmed by using the *B*. *subtilis* nitric oxide synthase bsNos as a metabolic target enzyme. Complementary mutational analyses confirmed that Fra acts as an essential component for maturation and subsequent targeting of the heme *b* co-factor, hence representing a key player in the iron-dependent physiology of *B*. *subtilis*.

## Introduction

Iron acts as an important and versatile redox element in most forms of life. It is the key component of a broad range of co-factors including iron-sulphur clusters (Fe-S) and heme, which are found in virtually all electron transport chains, in a vast number of oxidoreductases as well as in oxygen-binding and regulatory proteins [1,2]. However, free iron at high concentrations is responsible for the formation of reactive oxygen species and so can contribute to cytotoxicity [3]. Due to the importance of minimising the amount of free intracellular iron, highly efficient mechanisms have evolved for the protected transfer of this metal to its metabolic target and storage sites [4]. The protein frataxin is proposed to be associated with the function of intracellular iron homeostasis and trafficking. Generally, frataxin is a highly conserved mitochondrial protein in eukaryotes, and structural homologues are present in a number of bacteria [5,6]. Several studies have shown that a deficiency of frataxin in humans is the cause of Friedreich’s ataxia, a disease that affects the nervous system [7]. It has been demonstrated for several homologues that frataxin can serve as an iron chaperone which binds ferrous and ferric iron in relatively defined stoichiometries [5,8–11]. Previous reports have shown that bacterial frataxins, including *Escherichia coli* CyaY and *B*. *subtilis* Fra, are closely associated with the biogenesis of Fe-S, either in a regulatory way or by acting as an iron donor [11–16]. Yeast frataxin (Yfh1) forms physical complexes with both Fe-S scaffold proteins (IscU (bacteria), Isu1 (yeast)) and cysteine desulphurases (IscS (bacteria), ISD11/Isd11 (eukaryotes)) [17–19], and may also serve as a specific iron donor for these Fe-S assembly systems [20,21].

In addition to Fe-S assembly, frataxin was shown to interact with ferrochelatase homologues in yeast and human [22,23]. Ferrochelatase catalyses the insertion of ferrous iron into protoporphyrin IX to generate heme *b*, a common heme co-factor and precursor for all further heme derivatives [24,25]. Hence, it was suggested that frataxin is also involved in cellular heme biogenesis [26]. Indeed, it was observed that frataxin-deficient yeast cells are no longer able to supply sufficient amounts of iron to ferrochelatase for the formation of heme [22,27], and that overproduction of frataxin, on the other hand, stimulated heme biosynthesis at the expense of ISC assembly or stability [28]. Similarly, mitochondrial frataxin seems to be essential for heme biogenesis in *Arabidopsis* [29].

**Fig 1:**
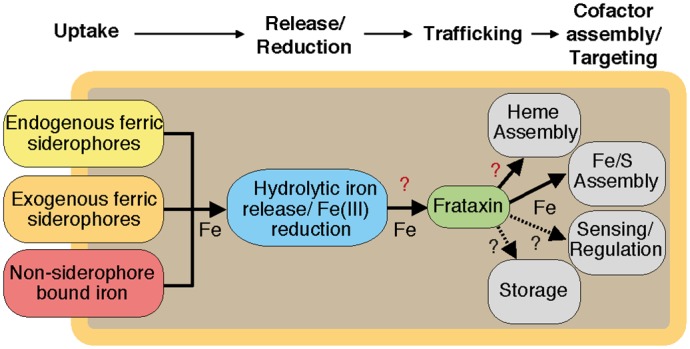
Overview of the proposed iron uptake and distribution pathways in *B*. *subtilis*. Iron is taken up either in the form of ferric siderophore complexes by transporters specific for endogenous or exogenous ferric siderophores [30,31], or in form of non-siderophore bound iron [32]. Iron in the cytosol is released from the siderophore complexes either in the ferric or ferrous state [30], and may undergo complete reduction until it is transferred to the iron chaperone frataxin (Fra) for intracellular trafficking to iron co-factor biogenesis systems, including iron-sulphur cluster (Fe-S) assembly [11] or, as characterised in this study, heme assembly. Further possible interactions with iron sensors/regulators or iron storage components have not yet been addressed yet.

We have recently reported that the structural frataxin homologue Fra of the Gram-positive soil bacterium *B*. *subtilis* plays a key role in Fe-S biogenesis [11]. The formation of Fe-S on the scaffold protein SufU was dependent on Fra, and a *fra* deletion had severe influence on cell growth and global iron homeostasis, raising the question whether frataxin might fulfil additional functions in the iron homeostasis network of *B*. *subtilis* (Fig. 1). In this study, we investigated the role of *B*. *subtilis* Fra in heme biogenesis and present, for the first time, detailed structural insights into the nature of interactions that are formed between Fra and HemH. In summary, we provide *in vitro* and *in vivo* evidence that *B*. *subtilis* frataxin is critical for heme *b* biogenesis and, consequently, provides a vital function for the global metabolism of this model bacterium.

## Material and Methods

### Co-purification experiments

In order to identify new potential Fra interaction partners, we generated a *B*. *subtilis* 168 complementation mutant carrying a xylose-inducible copy of His_6_-tagged *fra* in the *amyE* site (AM09), as described in [11], and carried out a co-purification. For this purpose, *B*. *subtilis* 168 AM09 cells and wild-type (WT) cells as a controll were grown in 100 mL LB medium to an OD_600_ of 0.6, harvested by centrifugation (16,000 x g, 4°C), resuspended in 2 mL buffer (50 mM HEPES, 300 mM NaCl, pH 8.0), and disrupted by using a FastPrep-24 instrument (MP Biomedicals). Cell debris was removed by centrifugation (16,000 x g, 4°C) and 2 mL crude protein extract was applied to a Qiagen Ni^2+^-NTA spin column, which was previously equilibrated with lysis buffer (50 mM NaH_2_PO_4_, 300 mM NaCl, 10 mM imidazole, pH 8.0). After loading, the column was washed twice with 600 μl wash buffer (50 mM NaH_2_PO_4_, 300 mM NaCl, 20 mM imidazole, pH 8.0) followed by elution of the proteins with 150 μl elution buffer (50 mM NaH_2_PO_4_, 300 mM NaCl, 500 mM imidazole, pH 8.0). The elution fractions were applied to sodium dodecyl sulfate polyacrylamide gel electrophoresis (SDS-PAGE), and the resulting bands of co-purified proteins were cut out and subjected to in-gel tryptic digestion and peptide mass fingerprinting. The WT crude protein extract experiments did not show any unspecific interactions. The experiments were repeated by immobilizing heterologesly expressed purified His_6_-tagged Fra onto the spin column, and heterologesly expressed purified StrepII-tagged HemH as an interaction partner. Control incubations with bovine serum albumin that were applied to a frataxin-loaded column were carried out in the same manner.

### MS-based protein identification and quantification

Samples were digested by the addition of Sequencing Grade Modified Trypsin (Promega) and incubated at 37°C overnight.

The mass spectrometric analysis of the samples was performed using an Orbitrap Velos Pro mass spectrometer (ThermoScientific). An Ultimate nanoRSLC-HPLC system (Dionex), equipped with a custom 20 cm x 75 μm column filled with 1.7 μm C18 beads, was connected online to the mass spectrometer through a Thermo Scientific Nanospray Flex Ion Source. 1 μL (4 μg protein crude extract) of the tryptic digest was injected onto a C18 pre-concentration column. Automated trapping and desalting of the sample was performed at a flowrate of 6 μL/min using water/0.05% formic acid as solvent.

Separation of the tryptic peptides was achieved with the following gradient of water/0.05% formic acid (solvent A) and 80% acetonitrile/0.045% formic acid (solvent B) at a flow rate of 300 nL/min: holding 4% B for five min, followed by a linear gradient to 45% B within 195 min and linear increase to 95% solvent B in additional 10 min. The column was connected to a stainless steel nanoemitter (Thermo Scientific) and the eluent was sprayed directly towards the heated capillary of the mass spectrometer using a potential of 2300 V. A survey scan with a resolution of 60000 within the Orbitrap mass analyzer was combined with at least ten data-dependent MS/MS scans with dynamic exclusion for 30 s using HCD-fragmentation combined with orbitrap detection at a resolution of 7500.

Data analysis was performed using Proteome Discoverer 1.4 (ThermoScientific) with SEQUEST and MASCOT (version 2.2; Matrix science) search engines using current SwissProt or NCBI databases with taxonomy *B*. *subtilis*.

For protein quantification the areas of identified peptides were normalized to the area of cytochrome c, which was added as an internal standard to the sample.

### Cloning, expression and purification of recombinant proteins

The bacterial strains and plasmids used in this study are listed in S1 and S2 Tables. The genes encoding Fra, HemH, FfoR, and bsNos were amplified via polymerase chain reaction from genomic DNA extracted from *B*. *subtilis* 168 using either the Invitrogen Platinum Pfx or the NEB Phusion High-Fidelity DNA Polymerase, according to the manufacturer’s protocol. The DNA oligonucleotides used for amplification are listed in S3 Table. Expression constructs for C-terminal Strep-tag II or N-terminal His_6_-tag fusions were constructed by using appropriate DNA restriction enzymes and subsequent DNA ligation [33]. The designed Strep-tag II expression constructs of *hemH* and *ffoR*, and the His_6_-tag expression constructs of *fra* and *nos* were used to transform *E*. *coli* BL21(DE3) cells, as described previously [33,34]. Expression and purification of the His_6_-tag proteins followed the procedures described in [35]. The manufacturer’s protocols of the pASK-IBA3 vector were used in the case of Strep-tag II expression constructs. In addition, all proteins were further subjected to size-exclusion chromatography on an ÄktaPurifier system (Amersham Pharmacia Biotechnology) by using a GE Healthcare preparative Superdex 75 26/60 column. All purified proteins were finally analysed by SDS-PAGE (S1 Fig.).

### Ultraviolet-visible (UV-Vis) spectroscopy

UV-Vis spectroscopic analyses of FfoR and Nos were carried out on an Ultrospec 3100 *pro* spectrophotometer (Amersham Pharmacia Biotechnology) using 1 cm path length quartz cuvettes. *Bacillus subtilis* nitric oxide synthase ligands were addressed using the separated monomeric apo- and dimeric holo-form of the protein. Difference spectra were recorded at 25°C between 300 and 800 nm in TRIS buffer W (25 mM TRIS/HCl, 150 mM NaCl, pH 8.0) either with or without 1 mM sodium dithionite. The investigation of purified FfoR was carried out in the same way.

### Fluorescence spectroscopy

Fluorescence spectroscopy was performed on a FP-6500 spectrofluorimeter (Jasco). Spectra were recorded by exciting the protoporphyrin IX soret band at 410 nm for the *in vitro* conversion of protoporphyrin IX into heme *b* by Fra/HemH. The emission peak analysed was 630 nm. The bandwidth for the excitation and emission was set to 5 nm, the sensitivity to medium and the response to 0.5 s. The heme emission band at 450 nm was analysed upon soret band excitations at 380 nm for the estimation of the heme concentration in crude cell extract. In this case, the bandwidth for the excitation and emission was set to 3 nm, the sensitivity to high and the response to 0.5 s. All spectra were recorded at 20°C.

### Chromatographic protein-protein interaction studies

Protein-protein interaction studies of Fra with HemH were carried out by analytical size-exclusion chromatography. An amount of 100 μM of each protein was incubated together in 1 mL TRIS buffer W (25 mM TRIS, 150 mM NaCl, pH 8.0) at 25°C for 30 min. Afterwards, 50 μL of the solution was applied to a GE Healthcare Superdex 200 10/300 GL column, previously calibrated with aldolase (158 kDa), ovalbumin (43 kDa), ribonuclease (13.7 kDa), and aprotinin (6.5 kDa), on an ÄktaPurifier system (Amersham Pharmacia Biotechnology). The elution with TRIS buffer W was monitored at 280 nm, and the elution volume was recorded for each peak. The apparent molecular weights of the eluted species were then calculated by using a molecular weight standard curve based on the protein calibration performed previously. The interactions were additionally investigated by co-purification with purified proteins. For this purpose, 2.5 nmol of His_6_-tagged Fra in HEPES buffer A (50 mM HEPES, 300 mM NaCl, pH 8.0) was immobilised on a Qiagen Ni^2+^-NTA spin column, which was subsequently treated with 50 μL of a 100 μM solution of Strep II-tagged HemH. Washing and elution were carried out according to the manufacturer’s protocol. Control incubations with bovine serum albumin that was applied to the frataxin-loaded column were carried out in the same manner. The experiments were repeated without immobilized Fra to exclude unspecific interactions of the Strep II-tagged proteins with the Ni^2+^-NTA spin column, but no unspecific interactions were found. The elution fractions where applied to SDS-PAGE for analysis (S2 Fig.).

### Microscale thermophoresis

Microscale thermophoresis was conducted for the determination of the Fra/HemH dissociation constant by using a Monolith NT.115 instrument (NanoTemper). Freshly prepared holo-Fra (80 μM in labelling buffer) was labelled at 15°C overnight using the cysteine reactive Monolith NT.115 Protein Labeling Kit RED (NanoTemper) to achieve a 1:1 molar ratio of labelled protein to dye. A serial titration from 42.6 μM to 0.01 μM of HemH in TRIS-based NT binding analysis buffer was prepared and labelled holo-Fra was diluted to each sample until a final concentration of 50 nM. The assay was then transferred into NT.115 enhanced gradient hydrophilic capillaries (NanoTemper) and measured at 25°C. The LED power for each measurement was set to 35% and the laser power to 40%. The heating time was set to 30 s, followed by 5 s of cooling. Binding curves were obtained from the thermophoresis hot to cold ratio (S3 Fig.). To investigate if the interaction is iron dependent the experiment was repeated using apo-Fra. Negative controls were performed with 50 nM labelled protein without the addition of HemH under the same conditions mentioned above. Each experiment was repeated in triplicate and an average *K*_d_ value was calculated afterwards with the NT Analysis software (NanoTemper).

### **Hydrogen**–**deuterium exchange**

Hydrogen–deuterium exchange (HDX) experiments were carried out on a custom-built liquid chromatography–mass spectrometry (LC-MS) set-up with a pepsin column, as described previously [36,37]. For the exchange reaction, either 250 nmol protein alone, or 250 nmol of each interaction partner in TRIS buffer W (25 mM TRIS, 150 mM NaCl, pH 8.0) was diluted ~1:50 in D_2_O exchange buffer (20 mM HEPES, 200 mM NaCl, 20 mM KCl, 20 mM MgCl_2_, pH 7.5) to a final volume of 50 μL and incubated for 0, 15, 30, and 60 s at 37°C. After incubation, the reaction was quenched with 50 μL quenching buffer (400 mM KH_2_PO_4_/H_3_PO_4_, pH 2.2) and injected directly into the LC-MS unit. All experiments were carried out in duplicate. After measurement and data acquisition, the HDX was determined using HDX Workbench software (S4 Fig.) [38] and mapped to crystal structures of *B*. *subtilis* Fra (Protein Databank ID code 2OC6) and HemH (Protein Databank ID code 2HK6) [39].

### HemH ferrochelatase activity

The holo-Fra-dependent kinetic reaction parameters of the conversion of protoporphyrin IX to heme *b* by HemH were determined as follows. At first, 2.7 μmol protoporphyrin IX were dissolved in 25 mL 100 mM TRIS, pH 7.2, containing 5% Tween 20. The exact concentration of protoporphyrin IX in solution was then determined via UV-Vis spectroscopy at 408 nm by using the extinction coefficient of 262 mM^-1^ cm^-1^. Recombinant apo-Fra (100 μM in reconstitution buffer) was loaded with iron by anaerobic incubation with FeCl_2_ (500 μM in reconstitution buffer) for 1 h. After incubation, excess iron was removed by gel filtration over an Econo-Pac 10DG desalting column (BioRad). The fractions containing protein were identified by quantitative Bradford assay [40]. Reactions were carried out in 1 mL reaction volume and contained 100 mM TRIS, pH 7.2, 50 μM holo-Fra and 0.1 μM to 10 μM protoporphyrin IX. Reactions were started under anaerobic conditions by adding 0.2 μM HemH, followed by anaerobic incubation at 25°C for 2 min, and were quenched by the addition of 100 μL of methanol. The reaction mixture was transferred into a 1 cm path length quartz cuvette and was analysed by fluorescence spectroscopy, as described previously. Control reactions were carried out with the single components and their different combinations with and without Fe^2+^-charged Fra. No conversion was observed without the addition of HemH.

### *In vitro* activity of the *B*. *subtilis* nitric oxide synthase bsNos

The *in vitro* activity of bsNos was assayed in 50 μL reactions containing the following: 10 μM holo-FfoR, 8 μM Ferredoxin from *Spinacia oleracea*, 1 mM NADPH, 2 μM tetrahydrofolic acid, 2 μM CaCl_2_, 2 μM FAD, 2 μM FMN, and 500 μM *N* ^ω^-hydroxy-l-arginine. Reactions were started by the addition of 2 μM of holo-bsNos and analysed by a Griess assay (Promega), according to the manufacturer’s protocol, at different time points. Control reactions were performed with the apo-form of bsNos and without the addition of bsNos. The amounts of nitric oxide generated were determined by measuring the concentration of its stable breakdown product nitrite using a standard calibration curve recorded within a nitrite concentration range of 1.56 to 50 μM.

### Investigation of the heme *b* co-factor targeting efficiency in crude cell extracts

An Δ*fra* deletion mutant was used as a reference strain with deleted frataxin [11]. *Bacillus subtilis* 168 and *B*. *subtilis* Δ*fra* cells were grown in 300 mL LB medium at 37°C to an OD_600_ of 0.5. The cells were pelleted, resuspended in 1 mL buffer containing 50 mM TRIS, 100 mM, NaCl, pH 7.4, and disrupted by using an MP Biomedicals FastPrep-24 instrument. Cell debris was removed by centrifugation (16,000 x g, 4°C) and the clear lysates were used for bsNos activity measurements, according to the procedure described above, by using crude protein extract (~ 1.5 mg) from the *B*. *subtilis* 168 WTcells or *B*. *subtilis* Δ*fra* cells. Control reactions were performed without the addition of *N* ^ω^-hydroxy-l-arginine. Nitric oxide production was then quantified by a Griess assay (Promega), according to the manufacturer’s protocol, at different time points. *In vivo* protein levels of bsNos in the WT and in the mutant were checked by tryptic digestion and quantitative peptide mass fingerprinting of respectively 4 μg crude protein extract.

### Estimation of the total intracellular protoporphyrin IX and heme content in *B*. *subtilis* WT and Δfra mutant cells

Cells were grown in 500 mL LB medium to an OD_600_ of 0.5, harvested by centrifugation (16,000 x g, 4°C), resuspended in 1 mL buffer (50 mM TRIS, 100 mM, NaCl, pH 7.4), and disrupted by using a FastPrep-24 instrument (MP Biomedicals). Cell debris was removed by centrifugation (16,000 x g, 4°C) and the total protein concentrations were determined by quantitative Bradford assay [40]. An amount of 350 μg of crude protein extract was diluted to 200 μL in ddH_2_O and extracted with 200 μL acidic acetone (20% 1.6 M HCl v/v). Precipitate was removed by centrifugation (16,000 x g, 4°C) and the supernatant was analysed by fluorescence spectroscopy, as described above. Assays were repeated twice and averages of the relative heme amounts determined were plotted together with their standard error of the mean (SEM).

### Anaerobic work

Anaerobic work was performed in an anaerobic chamber (Coy Laboratories) with forming gas (95% N_2_/5% H_2_) atmosphere. Solutions were made anoxic by purging with forming gas for at least 10 h. Solutions and plastic ware were allowed to equilibrate > 6 h inside the chamber before usage.

## Results and Discussion

### Frataxin acts as an intracellular ferrous iron donor for the ferrochelatase HemH

**Fig 2:**
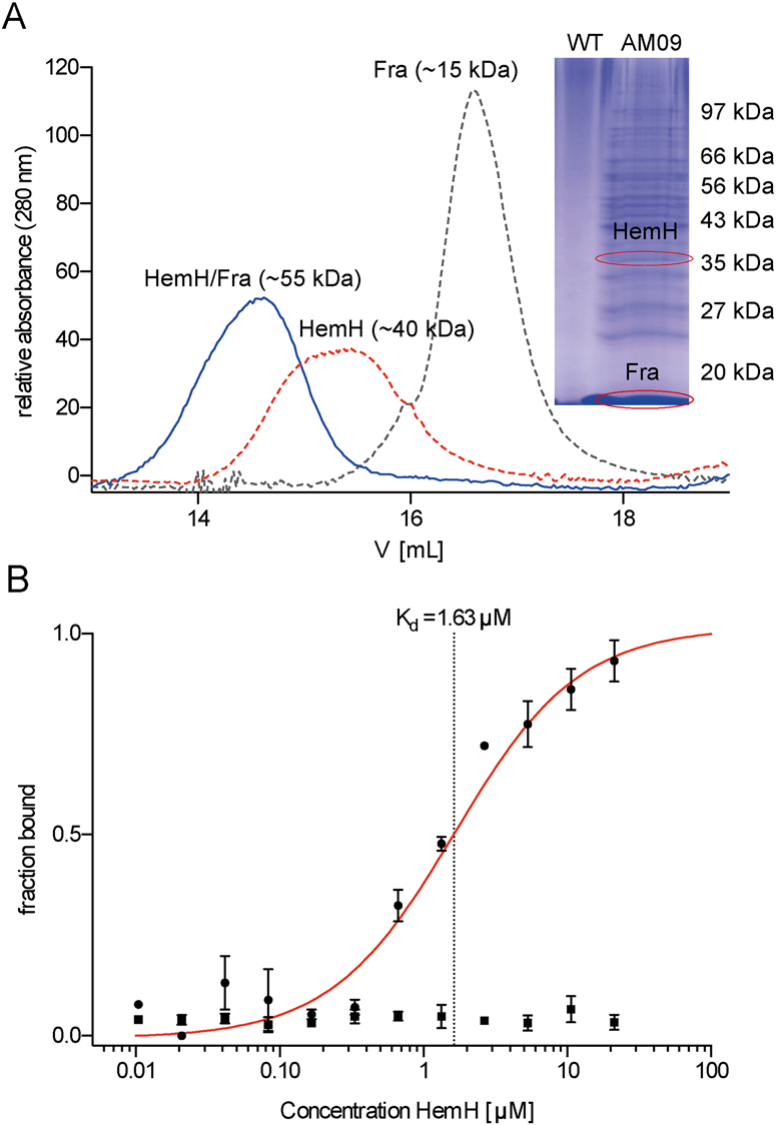
Interaction studies of frataxin (Fra) and ferrochelatase (HemH). (A) Ultraviolet-visible chromatogram of three independent analytical size-exclusion chromatography runs of heterologously expressed and purified Fra-His_6_ (gray dotted line), HemH-Strep II (red dotted line) and both proteins together, revealing the formation of a HemH/Fra interaction complex (blue solid line). The interactions were analysed by using a calibrated Superdex 200 10/300 GL gel-filtration column. The inset shows an SDS-PAGE of the Ni^2+^-NTA elution fraction of *B*. *subtilis* WT (WT) crude cell extract and *B*. *subtilis* AM09 (AM09) crude cell extract with endogenously expressed Fra-His_6_, which was co-purified with several proteins, including ferrochelatase HemH from *B*. *subtilis* AM09 crude cell extract. Control experiments did not reveal any unspecific interactions. (B) Fra/HemH dimerization was analysed thermophoretically. Unlabelled HemH was titrated to a constant amount of fluorescent-labelled apo-Fra (squares) and holo-Fra (circles). Dimerization caused a significant change in thermophoresis and a *K*_d_ of 1.63 μ 0.02 μM was calculated (red line). Error bars represent SEM of three independent experiments.

**Fig 3:**
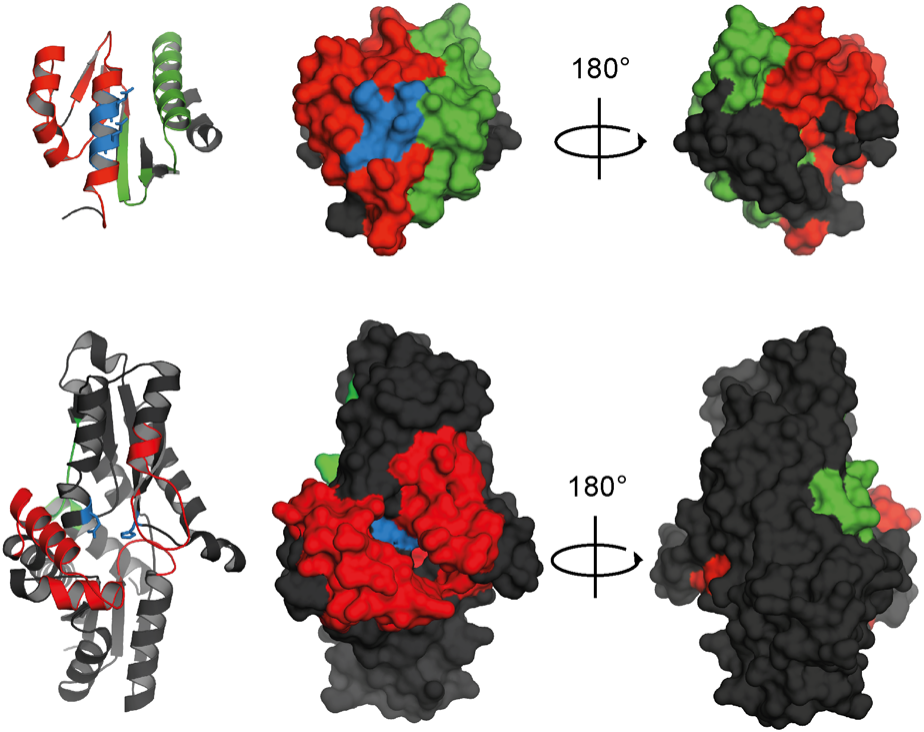
HDX epitope mapping of the Fra/HemH interaction surface. The acidic ridge of Fra (top) binds directly above the HemH (bottom) iron co-ordination site in its catalytic centre (blue), leading to a reduction in HDX (red). Upon binding, both enzymes undergo a conformational change, which leads either to an increased (green) or a reduced (red) HDX. Black areas indicate no change in HDX. The results were mapped to crystal structures of *B*. *subtilis* Fra (Protein Databank ID code 2OC6) and HemH (Protein Databank ID code 2HK6) [39].

**Fig 4:**
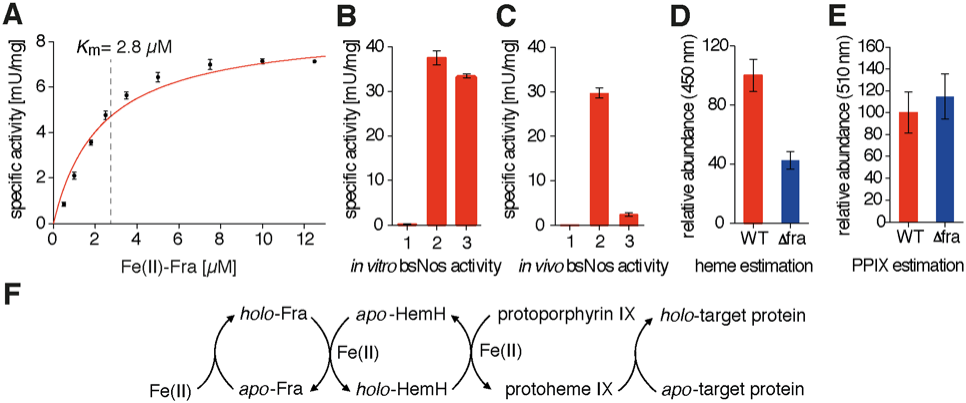
Influence of Fra on the heme maturation pathway. (A) Kinetics of the conversion of protoporphyrin IX into heme *b* by HemH. Varying concentrations of Fe^2+^-charged frataxin as the sole iron source were added in a range of 0.1–10 μM, and conversion of protoporphyrin IX into heme *b* was followed by fluorescence spectroscopy. Data were fitted according to the Michaelis-Menten model, which resulted in a *K*_m(obs)_ of about 2.8 ± 0.5 μM and a *k*_cat(obs)_ of 0.925 ± 0.059 s^-1^ (error bars represent SEM of three independent experiments). (B) *In vitro* activities of apo-bsNos (1), holo-bsNos (2) and reconstituted apo-bsNos (3). The reconstitution of apo-bsNos with its heme co-factor was carried out by a coupled transfer assay containing Fe^2+^-charged Fra, HemH and protoporphyrin IX, and led to a partial restoration of bsNos activity (error bars represent SEM of three independent experiments). (C) *In vivo* bsNos activities in equalized amounts of total cellular protein. Control of the assay with *B*. *subtilis* WT crude protein extract assayed without the addition of *N* ^ω^-hydroxy-l-arginine (1), with *B*. *subtilis* WT crude protein extract (2) and with *B*. *subtilis* Δ*fra* crude protein extract (3). The deletion of *fra* led to a ~12-fold decrease of bsNos activity in the crude mutant cell extract (error bars represent SEM of three independent experiments). (D) Determination of the relative heme contents in *B*. *subtilis* WT (red bar) and Δ*fra* (blue bar) cells by acidic acetone extraction and fluorescence analysis of the heme *b* soret band emission at 450 nm upon excitation at 380 nm. The amount of cellular heme was found to be ~2.5-fold reduced in the Δ*fra* mutant cell (error bars represent SEM of three independent experiments). (E) Determination of the relative protoporphyrin IX concentration in *B*. *subtilis* WT (red bar) and Δ*fra* (blue bar) cells by acidic acetone extraction and fluorescence analysis of the protoporphyrin IX soret band emission at 510 nm upon excitation at 410 nm. The amount of cellular protoporphyrin IX was ~1.2-fold elevated in the Δ*fra* mutant cell (error bars represent SEM of two independent experiments). (F) Fra mediated heme *b* (protoheme IX) maturation and its delivery to heme-dependent target proteins.

In order to identify novel interaction partners of frataxin within the cellular network of iron homeostasis, we generated a *B*. *subtilis* 168 complementation mutant carrying a xylose-inducible copy of His_6_-tagged *fra* in the *amyE* site. Co-purification and peptide mass fingerprinting revealed the ferrochelatase HemH as a potential interaction partner of Fra (Fig. 2A). In addition to HemH the SDS-PAGE showed several other protein bands that were also analysed by peptide mass fingerprinting. The identified proteins could represent further potential Fra interaction partners and are subject to future work. Protein-protein interaction studies were carried out using analytical size-exclusion chromatography to examine if Fe^2+^ -charged Fra (~17 kDa; produced as a His_6_-tag variant, S1 Fig.) and HemH (~40 kDa; produced as a Strep-tag II variant, S1 Fig.) specifically interact *in vitro*. The UV-Vis chromatogram of the elution profile showed a broader peak at ~55 kDa, leading to the conclusion that Fra and HemH form stable heterodimers (Fig. 2A). This conclusion was further supported by the results of an *in vitro* co-purification of the two proteins (S2 Fig.). However, no complexes of higher molecular weight were observed, probably due to the fact that the *B*. *subtilis* ferrochelatase is active in a monomeric form [24]. Using microscale thermophoresis, we were able to determine a *K*_d_ of 1.63 ± 0.02 μM for the holo-Fra/HemH interaction. The repetition of the experiment using apo-Fra did not lead to any observable complex formation in the μM range, indicating that the interaction is at least partially iron dependent (Figs. 2B and S3). Epitope mapping experiments were performed using HDX to gain detailed structural insights into the interaction topology, and revealed that the proposed iron-binding site of Fra [16] faces the HemH active site directly (Figs. 3 and S4). A closer investigation of the interaction site revealed the presence of mainly acidic and hydrophobic α-helices, which form a large interface of about 550 Å^2^. The mode of recognition appears to be a complex mixture of ionic and partially hydrophobic interactions. The Fe^2+^ ion is most likely bound to frataxin’s acidic ridge, which appears to reach into the iron-binding pocket of HemH and possibly transfers the iron to the His_183_ and Glu_264_ residues which are responsible for iron binding in HemH [39]. The HDX data further showed that the interaction leads to a significant conformational change in both proteins, during which HemH seems to clasp the acidic ridge of Fra, suggesting an induced fit mechanism for the supposed ferrous iron transfer during this interaction. Additional conformational changes that might be associated with the process of iron transfer and the changing apo/holo state of the two proteins may accompany the subsequent dissociation of the complex. This is, to the best of our knowledge, the first specific structural evidence for the interaction of frataxin with an iron-dependent protein of the heme biogenesis pathway. HemH utilises ferrous iron for the conversion of protoporphyrin IX into protoheme IX (heme *b*). The conversion of protoporphyrin IX into heme *b* was followed by fluorescence spectroscopy to test if a specific transfer of iron takes place during the interaction of holo-Fra with HemH. Indeed, a formation of heme *b* in the presence of Fe^2+^ -charged frataxin as the sole iron source was observed under anaerobic conditions. In order to determine the kinetic parameters of this frataxin-dependent reaction, the concentrations of iron-loaded holo-Fra were varied between 0.1 and 10 μM, and reactions were analysed within the linear range of the time-dependent conversion of protoporphyrin IX into heme *b* (S5 Fig.). The specific activities determined for the total reaction were fitted according to the Michaelis-Menten model, resulting in a *K*_m(obs)_ of about 2.8 ± 0.5 μM and a kcat(obs) of 0.925 ± 0.059 s-1 (Fig. 4A).

### The deletion of frataxin has a generally negative effect on heme biogenesis in *B*. *subtilis*

We used a Δ*fra* deletion mutant [11] and assayed the activity of heme-dependent nitric oxide synthase (bsNos) as a representative target enzyme to examine the effect of a frataxin deficiency on heme maturation *in vivo*. *B*. *subtilis* nitric oxide synthase is responsible for the generation of the cellular signalling molecule NO by the NADPH-dependent oxidation of l-arginine into l-citrulline and forms *N* ^ω^-hydroxy-l-arginine as a reaction intermediate [41]. Holo-bsNos contains heme *b* as a co-factor and is present as a homodimer, while the inactive apo-form is a monomer [42]. We first designed an *in vitro* assay with the recombinant His_6_-tagged variants of purified bsNos and FfoR, a Fur-regulated NADPH:ferredoxin oxidoreductase of *B*. *subtilis* (formerly YcgT) [43] with bound FAD co-factor (S6 Fig.), and observed the generation of NO by the utilisation of *N* ^ω^-hydroxy-l-arginine as a direct substrate. The separation of the apo- and holo-forms of bsNos was carried out by size-exclusion chromatography after affinity purification from the crude protein extract. As expected, only the dimeric holo-form of bsNos was able to generate NO in detectable amounts. In order to test if apo-bsNos could be reconstituted with its heme co-factor in a coupled transfer assay, we anaerobically incubated the monomeric species with Fe^2+^-charged Fra, HemH and protoporphyrin IX. Subsequently, *N* ^ω^-hydroxy-l-arginine was added, resulting in the production of significant amounts of NO, indicating that an *in vitro* reconstitution of the enzyme occurred due to utilisation of the heme *b* species generated (Fig. 4B). After establishing the detection of NO formation with purified proteins *in vitro*, we applied the assay to crude protein extracts of *B*. *subtilis* WT and Δ*fra* mutant cells. We found that the activity of bsNos in Δ*fra* mutant cells was ~12-fold decreased compared to the WT cells (Fig. 4C). To ensure that the decrease in activity is not due to a decrease of bsNos protein levels in the Δ*fra* deletion mutant we carried out quantitative peptide mass fingerprinting, which revealed that the deletion of *fra* does not lead to such (S7 Fig.). A quantitative analysis of intracellular heme and protoporphyrin IX contents was carried out by extraction with acidic acetone to test if this loss of heme enzyme activity was associated with a generally decreased heme content in the frataxin mutant [44]. The total amount of cellular heme was found to be ~2.5-fold lower in crude Δ*fra* mutant cell extracts compared to crude WT extracts based on equal amounts of total protein (Figs. 4D and S8). By contrast, the protoporphyrin IX ratio was ~1.2-fold higher in the Δ*fra* mutant compared to the WT (Figs. 4E and S8). Altogether, these results suggest that Fra represents a key component in the heme maturation pathway of *B*. *subtilis* by directing the iron trafficking process to the downstream-acting ferrochelatase HemH, which, subsequently, releases heme *b* that can be built into metabolic target enzymes (Fig. 4E).

**Fig 5:**
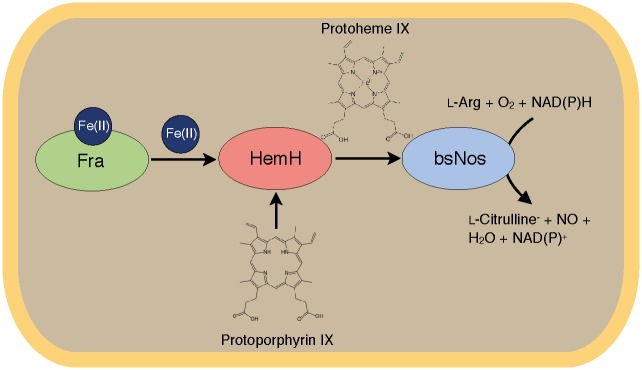
Scheme of the proposed iron channelling pathway. Ferrous iron is bound to the iron chaperone Fra, which transfers it by physical interaction to the ferrochelatase HemH. Upon incorporation of ferrous iron into the protoporphyrin IX scaffold by HemH, heme *b* is generated and can serve as a co-factor for heme-dependent target enzymes, such as nitric oxide synthase bsNos.

A number of previous studies have demonstrated that bacterial and eukaryotic frataxin homologues are capable of interacting with proteins and enzymes that are involved in Fe-S and heme biosynthesis [7,45]. However, the functional role of frataxin is still highly debated [1,12,46]. In this study, we present evidence that the *B*. *subtilis* frataxin homologue Fra can serve as an intracellular carrier to provide iron for the ferrochelatase HemH. Several studies suggest that frataxin participates directly in the cellular heme maturation pathway, although no physical interaction studies have been carried out so far [6,22]. Therefore, we thought to test whether Fra is able to interact directly with the *B*. *subtilis* ferrochelatase HemH in order to supply ferrous iron for the synthesis of heme *b*. Our results demonstrate that Fra indeed interacts with HemH and is able to supply iron to the HemH active site, probably via a mutually induced conformational change. This is, to the best of our knowledge, the first time that a physical interaction between a frataxin homologue and a heme biogenesis protein has been reported and that the protein-protein interface formed is topologically characterised. The current results from the *Bacillus* system are corroborated by observations in human cells and *S*. *cerevisiae*, where frataxin and its homologues are able to supply iron for ferrochelatase activity [5,22,23,27,47,48]. Interestingly, in contrast to human ferrochelatase, *B*. *subtilis* HemH does not contain an Fe-S cluster as a cofactor [24,25,49], hence it is unlikely that ISC assembly in *B*. *subtilis* interferes with its heme maturation pathway [50]. Furthermore, our *in vivo* experiments demonstrate that the deletion of *fra* leads to a reduced maturation of heme-dependent target enzymes, such as the nitric oxide synthase bsNos, as well as to a generally reduced cytosolic heme content accompanied by slightly elevated protoporphyrin IX levels. In fact, a similar effect of changed heme/protoporphyrin IX ratios has also been observed in frataxin-deficient patients suffering from Friedreich’s ataxia [7]. In conclusion, this study describes, with structural details, an intracellular frataxin-dependent iron trafficking pathway for the biosynthesis of the essential heme *b* co-factor and its subsequent supply to metabolic target enzymes (Fig. 5). These findings point to the general importance of frataxin to maintain fundamental physiological processes in the Gram-positive model bacterium *B*. *subtilis*.

## Supporting Information

S1 Fig SDS-PAGE of purified recombinant proteins used in this study. Fra-His_6_ (17.2 kDa), Nos-His_6_ (43.3 kDa), HemH-StrepII (39.6 kDa) and FfoR-StrepII (39.6 kDa). Molecular weights were calculated by using a calibration standard curve.
(TIF)

S2 Fig *In vitro* co-purification of HemH with immobilized Fra. Heterologously expressed and purified His_6_-tagged Fra was immobilized on a Qiagen Ni^2+^-NTA spin column and treated with a solution of heterologously expressed and purified StrepII-tagged HemH. The SDS-PAGE shows the elution fractions of a control done with BSA instead of HemH which shows that no unspecific binding occurs, a control done without immobilized Fra which shows that HemH alone does not interact with the Ni^2+^-NTA spin column (Ctrl), and the co-purification (Elu) of the Fra/HemH complex.
(TIF)

S3 Fig Microscale thermophoresis (MST). MST fluorescence curves of the Fra/HemH interaction with iron charged Fra (top) and without iron charged Fra (bottom) where recorded by measuring a serial titration of HemH with diluted labelled holo-Fra in NT.115 enhanced gradient hydrophilic capillaries (NanoTemper) at 25°C. The LED power for each measurement was set to 35% and the laser power to 40%. The heating time was set to 30 s, followed by 5 s of cooling. For *K*_d_ determination the hot (red lines) to cold (blue lines) ratio was taken.
(TIF)

S4 Fig Hydrogen-deuterium exchange (HDX)-MS experiments. (A) HDX-MS of Fra in complex with HemH. The D_2_O accessibility of backbone amides was determined by the percentage of amides exchanging with rates greater than 5 min^−1^ for each pepsin-generated peptide. The percentage exchanged for Fra alone was subtracted from the percentage obtained for the Fra/HemH complex. Green bars indicate an increase of HDX upon interaction, red bars a decrease. (B) HDX-MS of HemH in complex with Fra. The D_2_O accessibility of backbone amides was determined by the percentage of amides exchanging with rates greater than 4 min^−1^ for each pepsin- generated peptide. The percentage exchanged for HemH alone was subtracted from the percentage obtained for Fra/HemH complex. Green bars indicate an increase of HDX upon interaction, red bars a decrease.
(TIF)

S5 Fig Fra dependent heme *b* formation. (A) Fluorescence spectra of the conversion of protoporphyrin IX into protoheme IX (heme *b*) measured between 1 min (red line) and 6 min (blue line). The increasing emission peak at ~590 nm shows the formation of heme *b* over time, the decreasing peak at ~635 nm the consumption of protoporphyrin IX. (B) Time dependent conversion of protoporphyrin IX in the presents of apo-Fra (green line), Fe(II) charged holo-Fra (red line) and free Fe(II) (blue line).
(TIF)

S6 Fig Characterization of the ferredoxin oxidoreductase FfoR. UV-vis absorption spectra of oxidized (red line) and reduced (blue line) FfoR bound FAD.
(TIF)

S7 Fig MS-based bsNos quantification in WT and Fra deficient cells. The bsNos protein levels in the wild type (WT) and the Fra deficient (Δ*fra*) crude protein extract were investigated by tryptic digestion and quantitative peptide mass fingerprinting. The results show that both, the wild type and the mutant cell, share similar levels of bsNos. Error bars represent SEM of three independent experiments.
(TIF)

S8 Fig Relative heme *b* and protoporphyrin IX *in vivo* levels of Fra deficient cells. (A) Determination of the relative heme concentration at the emission wavelength 450 nm of the heme *b* soret band upon excitation at 380 nm. (B) Determination of the relative protoporphyrin IX concentration at the emission wavelength of 510 nm upon excitation at 410 nm.
(TIF)

S1 Table Bacterial strains used in this study. (TIF)

S2 Table Plasmids used in this study. (TIF)

S3 Table Oligonucleotides used for DNA amplification. (TIF)
