# Supplementary figures and images for: Molecular Insights into Frataxin-Mediated Iron Supply for Heme Biosynthesis in Bacillus subtilis
Source: PLoS One. 2015 Mar 31;10(3):e0122538. doi: 10.1371/journal.pone.0122538 (PMC4380498; doi:10.1371/journal.pone.0122538)

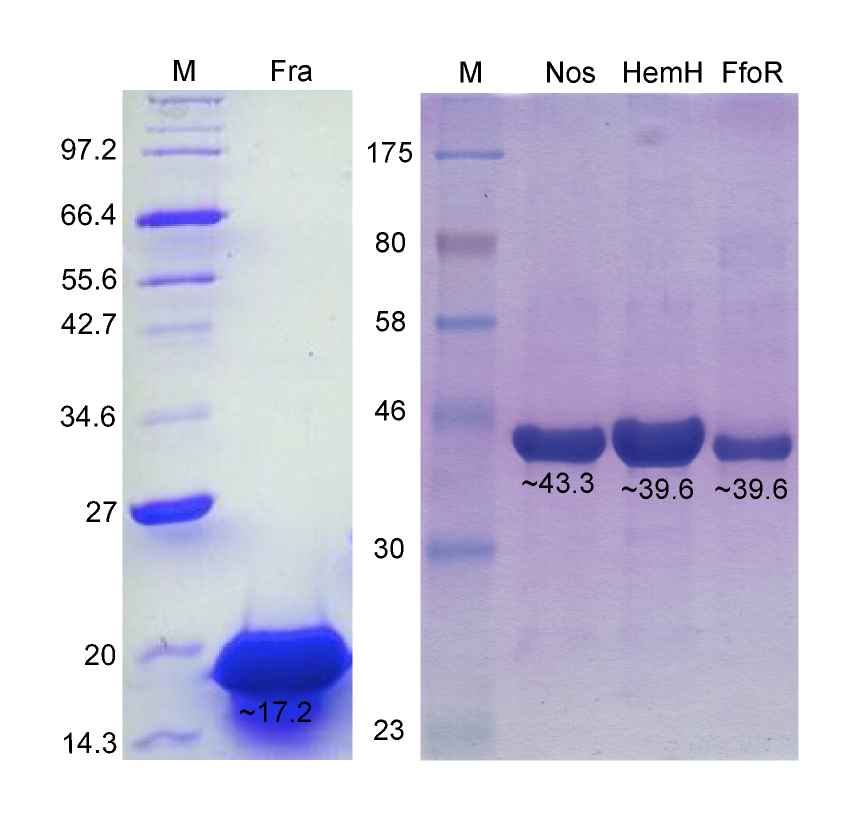

Supplement: S1 Fig — Fra-His6 (17.2 kDa), Nos-His6 (43.3 kDa), HemH-StrepII (39.6 kDa) and FfoR-StrepII (39.6 kDa). Molecular weights were calculated by using a calibration standard curve. (TIF) [file pone.0122538.s001.tif]

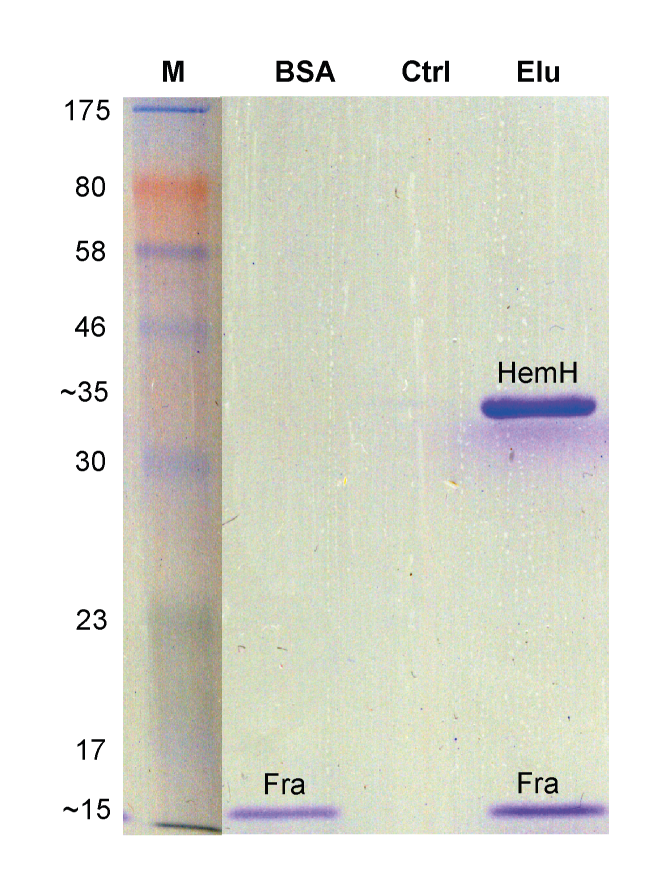

Supplement: S2 Fig — Heterologously expressed and purified His6-tagged Fra was immobilized on a Qiagen Ni2+-NTA spin column and treated with a solution of heterologously expressed and purified StrepII-tagged HemH. The SDS-PAGE shows the elution fractions of a control done with BSA instead of HemH which shows that no unspecific binding occurs, a control done without immobilized Fra which shows that HemH alone does not interact with the Ni2+-NTA spin column (Ctrl), and the co-purification (Elu) of the Fra/HemH complex. (TIF) [file pone.0122538.s002.tif]

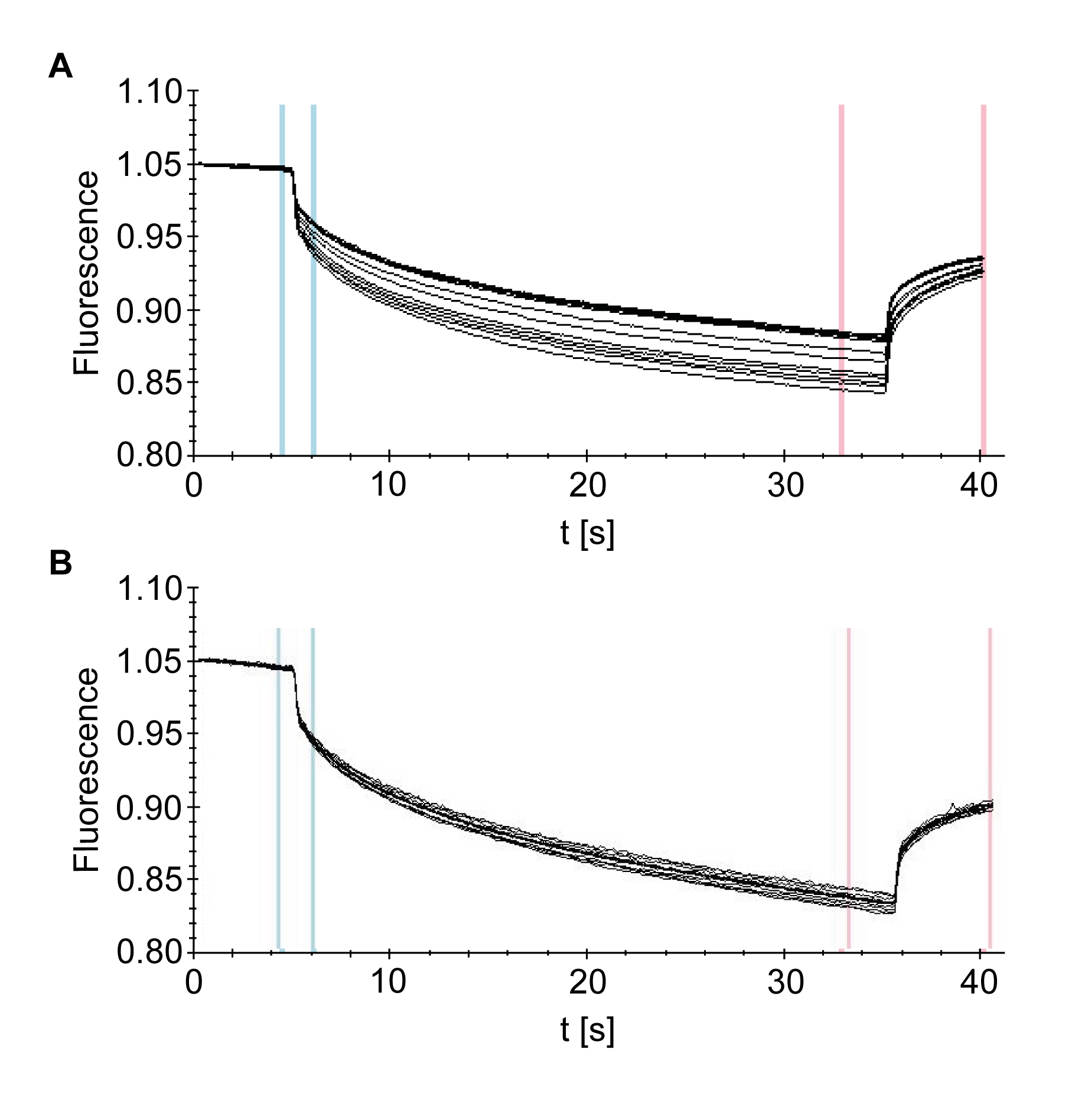

Supplement: S3 Fig — MST fluorescence curves of the Fra/HemH interaction with iron charged Fra (top) and without iron charged Fra (bottom) where recorded by measuring a serial titration of HemH with diluted labelled holo-Fra in NT.115 enhanced gradient hydrophilic capillaries (NanoTemper) at 25°C. The LED power for each measurement was set to 35% and the laser power to 40%. The heating time was set to 30 s, followed by 5 s of cooling. For K d determination the hot (red lines) to cold (blue lines) ratio was taken. (TIF) [file pone.0122538.s003.tif]

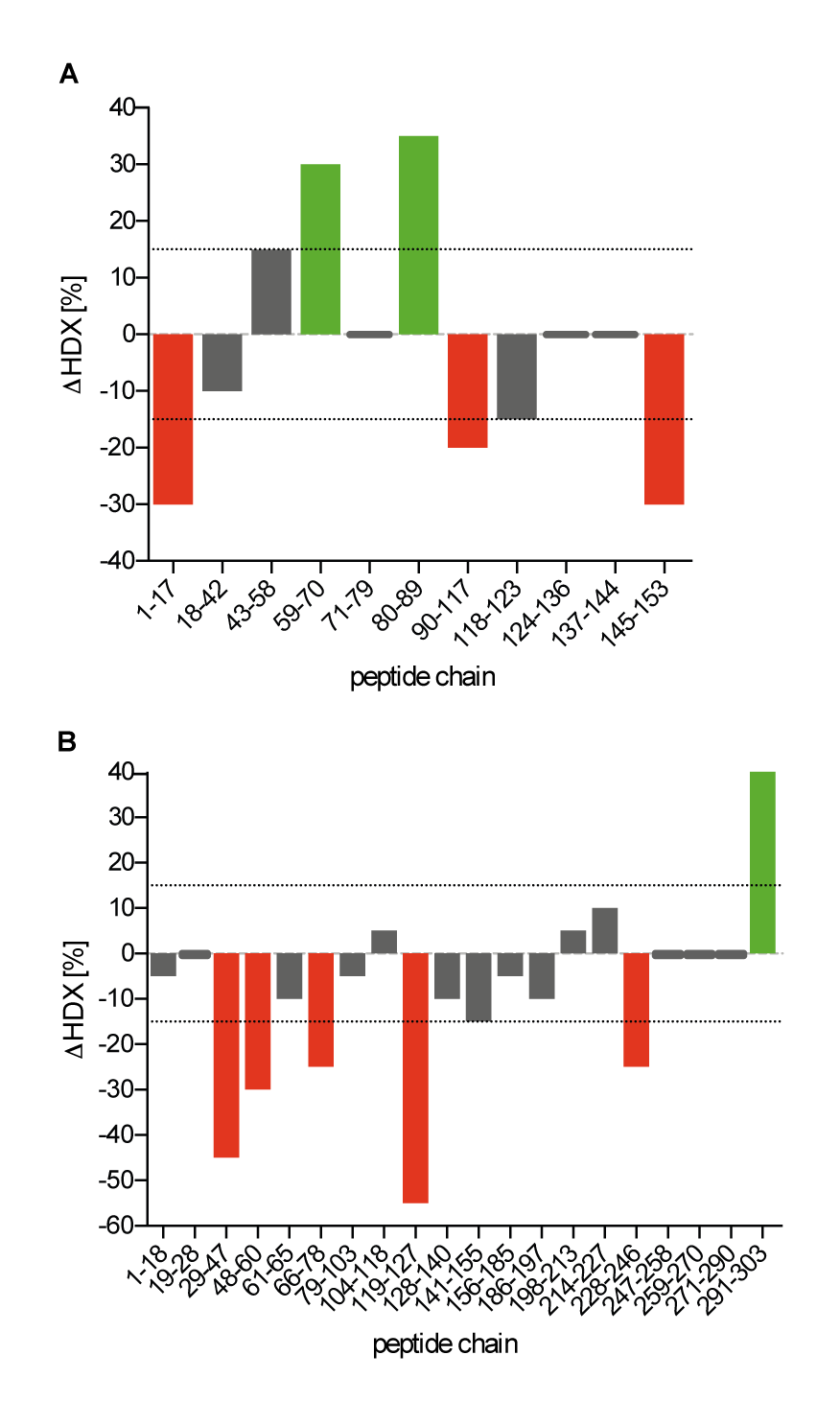

Supplement: S4 Fig — (A) HDX-MS of Fra in complex with HemH. The D2O accessibility of backbone amides was determined by the percentage of amides exchanging with rates greater than 5 min−1 for each pepsin-generated peptide. The percentage exchanged for Fra alone was subtracted from the percentage obtained for the Fra/HemH complex. Green bars indicate an increase of HDX upon interaction, red bars a decrease. (B) HDX-MS of HemH in complex with Fra. The D2O accessibility of backbone amides was determined by the percentage of amides exchanging with rates greater than 4 min−1 for each pepsin- generated peptide. The percentage exchanged for HemH alone was subtracted from the percentage obtained for Fra/HemH complex. Green bars indicate an increase of HDX upon interaction, red bars a decrease. (TIF) [file pone.0122538.s004.tif]

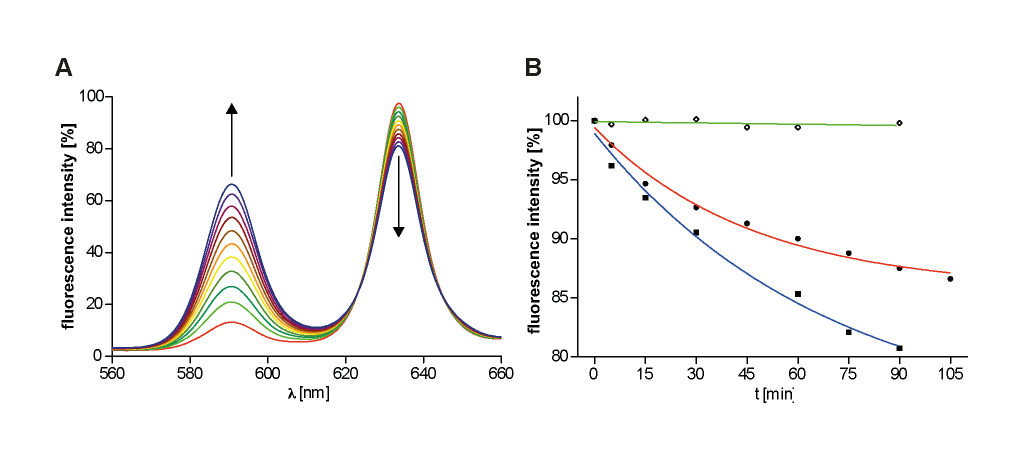

Supplement: S5 Fig — (A) Fluorescence spectra of the conversion of protoporphyrin IX into protoheme IX (heme b) measured between 1 min (red line) and 6 min (blue line). The increasing emission peak at ~590 nm shows the formation of heme b over time, the decreasing peak at ~635 nm the consumption of protoporphyrin IX. (B) Time dependent conversion of protoporphyrin IX in the presents of apo-Fra (green line), Fe(II) charged holo-Fra (red line) and free Fe(II) (blue line). (TIF) [file pone.0122538.s005.tif]

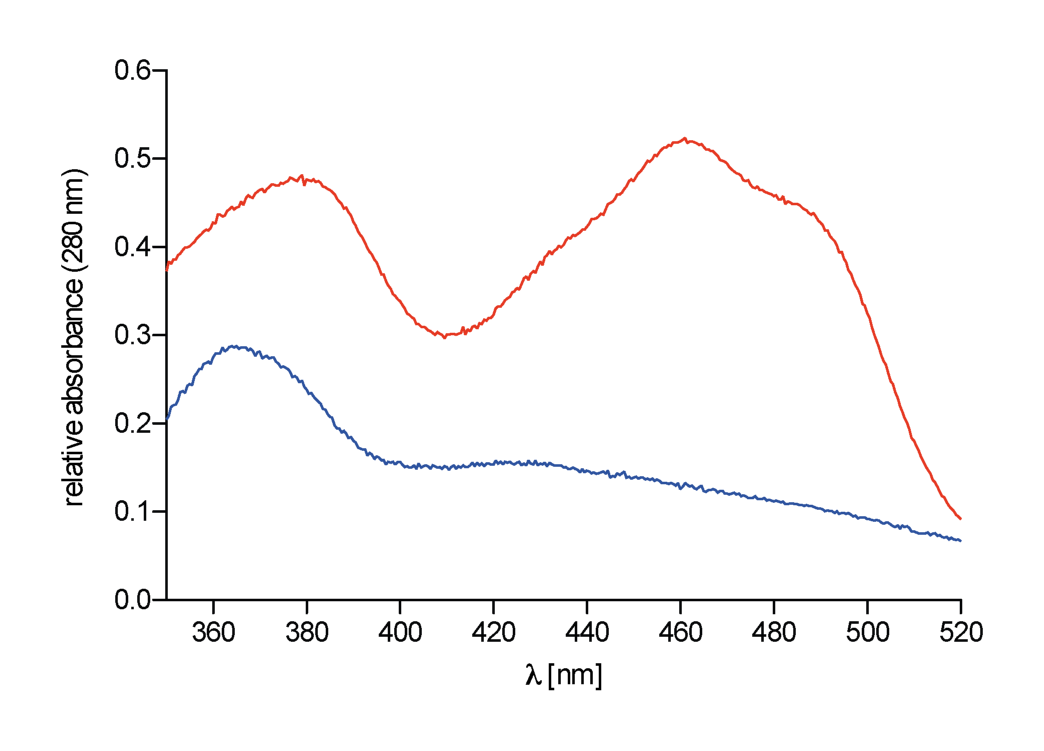

Supplement: S6 Fig — UV-vis absorption spectra of oxidized (red line) and reduced (blue line) FfoR bound FAD. (TIF) [file pone.0122538.s006.tif]

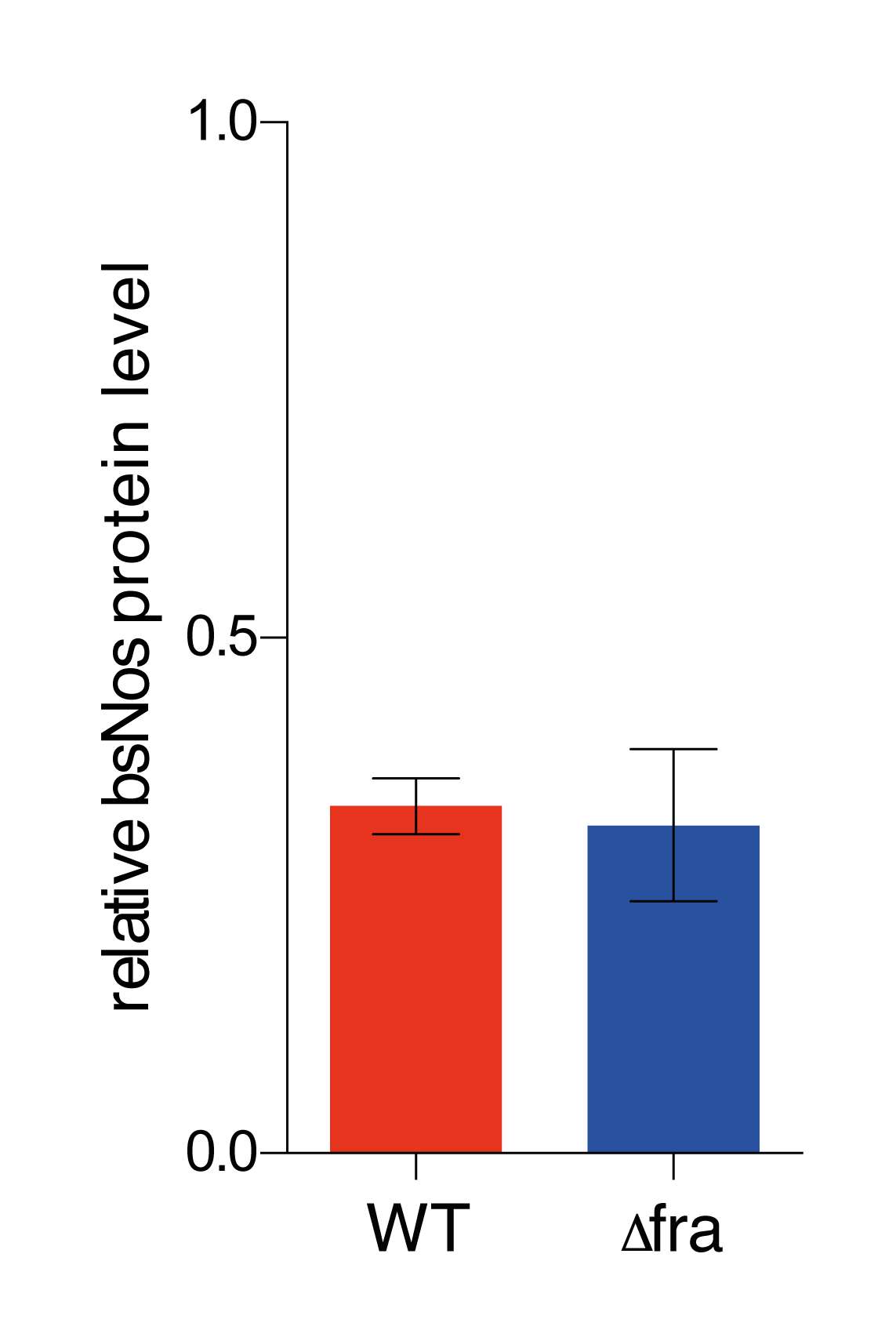

Supplement: S7 Fig — The bsNos protein levels in the wild type (WT) and the Fra deficient (Δfra) crude protein extract were investigated by tryptic digestion and quantitative peptide mass fingerprinting. The results show that both, the wild type and the mutant cell, share similar levels of bsNos. Error bars represent SEM of three independent experiments. (TIF) [file pone.0122538.s007.tif]

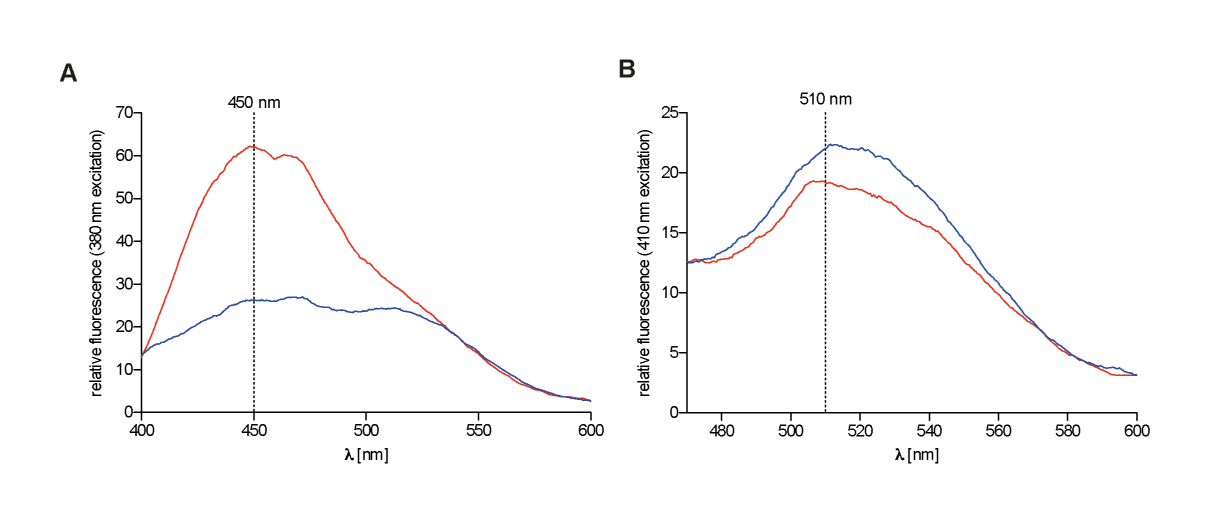

Supplement: S8 Fig — (A) Determination of the relative heme concentration at the emission wavelength 450 nm of the heme b soret band upon excitation at 380 nm. (B) Determination of the relative protoporphyrin IX concentration at the emission wavelength of 510 nm upon excitation at 410 nm. (TIF) [file pone.0122538.s008.tif]

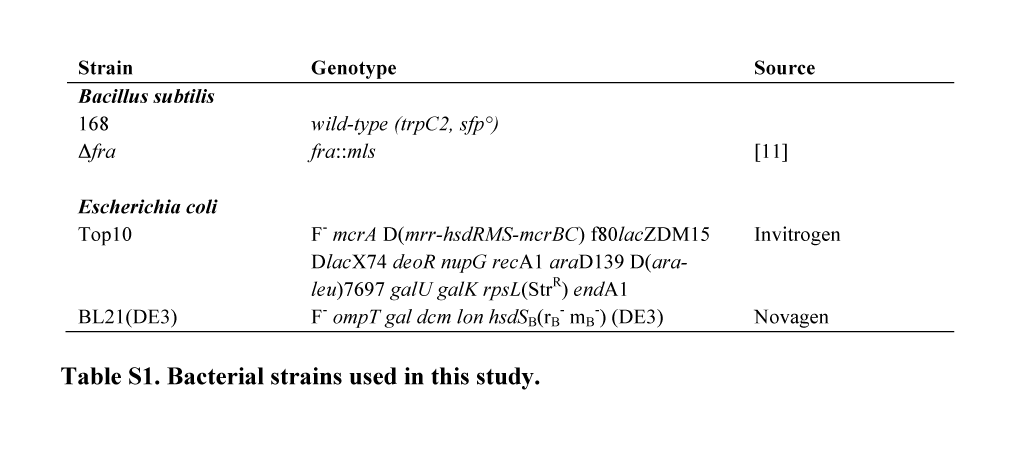

Supplement: S1 Table — (TIF) [file pone.0122538.s009.tif]

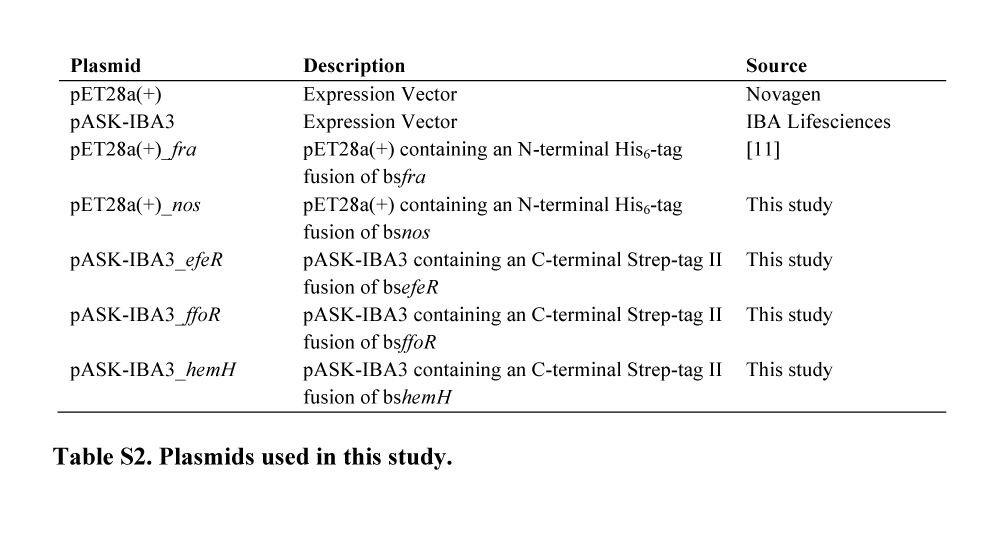

Supplement: S2 Table — (TIF) [file pone.0122538.s010.tif]

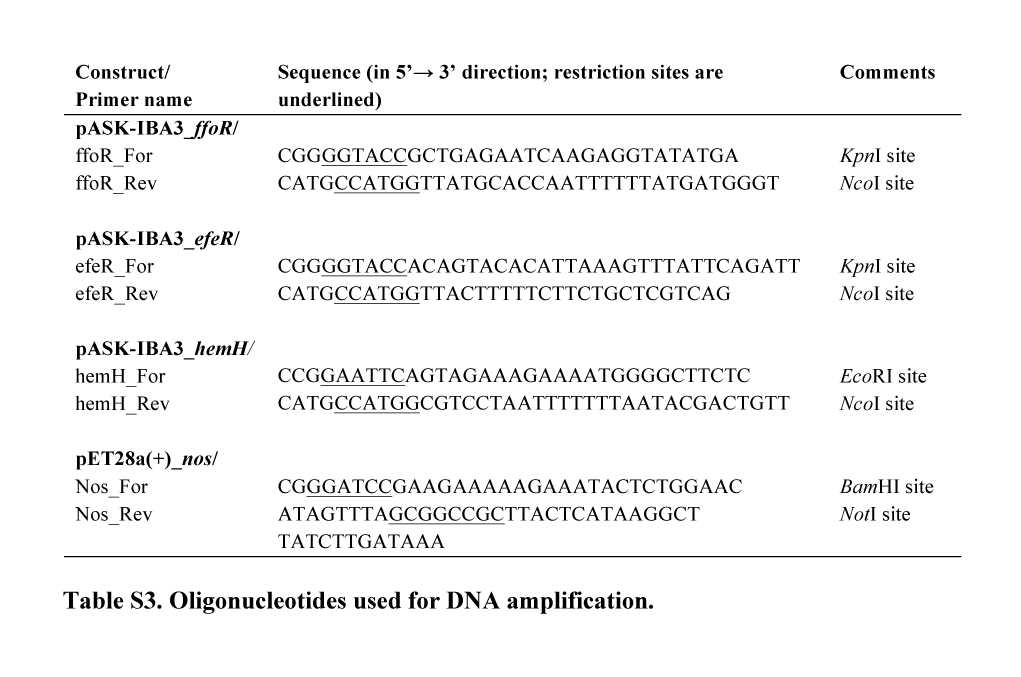

Supplement: S3 Table — (TIF) [file pone.0122538.s011.tif]
